# Preoperative chemoradiotherapy creates an opportunity to perform sphincter preserving resection for low-lying locally advanced rectal cancer based on an oncologic outcome study

**DOI:** 10.18632/oncotarget.10303

**Published:** 2016-06-27

**Authors:** Jun-Zhong Lin, Jian-Hong Peng, Aiham Qdaisat, Zhen-Hai Lu, Xiao-Jun Wu, Gong Chen, Pei-Rong Ding, Li-Ren Li, Yuan-Hong Gao, Zhi-Fan Zeng, De-Sen Wan, Zhi-Zhong Pan

**Affiliations:** ^1^ Department of Colorectal Surgery, Sun Yat-sen University Cancer Center, State Key Laboratory of Oncology in South China, Collaborative Innovation Center for Cancer Medicine, Guangzhou, 510060, P.R. China; ^2^ Department of Emergency Medicine, The University of Texas MD Anderson Cancer Center, Houston, TX, 77030, USA; ^3^ Department of Radiation Oncology, Sun Yat-sen University Cancer Center, State Key Laboratory of Oncology in South China, Collaborative Innovation Center for Cancer Medicine, Guangzhou, 510060, P.R. China

**Keywords:** preoperative chemoradiotherapy, sphincter preserving resection, rectal cancer, oncologic outcome

## Abstract

Low-lying locally advanced rectal cancer (LARC) after preoperative chemoradiotherapy (CRT) can be surgically removed by either abdominperineal resection (APR) or sphincter preserving resection (SPR). This retrospective cohort study of 251 consecutive patients with low lying LARC who underwent CRT followed by radical surgery in a single institute, between March 2003 and November 2012, aimed to compare the oncological benefits between the two groups. 3-year disease free survival (DFS), overall survival (OS), cumulative incidence of recurrence and postoperative complications were compared between the two approaches. With median follow-up of 48.6 months, SPR group had higher 3-year DFS rate (86.4% vs 73.6%, P=0.023) and lower incidence of distant recurrence (12.0% vs 23.7%, P=0.026). The postoperative complications, incidence of local recurrence and the 3-year OS were comparable between the two groups. Pathologic T and N stage were the independent predictors for 3-year DFS (P=0.020 and P<0.001). In conclusion, our study suggest that low-lying LARC patients with a significant response to preoperative CRT can benefit from the advantage of SPR in preserving the anal sphincter function without compromising their oncologic outcome.

## INTRODUCTION

Previously, abdominoperineal resection (APR) has been recognized as the preferred surgical method for patients with very low-lying rectal tumors [1]. Despite the fact that this procedure will successfully resect the tumor, it fails to preserve the anal sphincter resulting in the impairment of the anorectal function. Recently, with the development of comprehensive treatment, preoperative chemoradiotherapy (CRT) followed by total mesorectal excision (TME) has been the standard treatment pattern for locally advanced rectal cancer (LARC), which not only improves the local disease control but also preserves the integrity of the anal sphincter, hence preserving its function [2, 3]. Treated with preoperative CRT, most patients achieved a clinically meaningful tumor regression, even reaching a pathologic complete response (pCR) [4, 5]. Consequently, downstaging and downsizing the tumor by preoperative CRT allows the surgeon to perform a sphincter preserving resection (SPR) for patients whom APR was initially planned as their next step [6, 7]. In fact, patients who received SPR were reported to have higher quality of life than that of those who received APR according to better body image, more sufficient social and sexual functions [8–10]. Thereby, SPR was well accepted for both patients and surgeons, becoming significantly expanded, or even outweighing APR for treating lower ultra-lower rectal cancer. However, whether sphincter preservation surgery impairs disease control or oncologic survival after CRT in contrast to APR has not been well demonstrated [11, 12]. Due to this unclear oncologic survival after SPR, the decision regarding the selection of APR or SPR to treat low-lying LARC after CRT remains controversial.

To achieve a more definite result, we conducted a retrospective cohort study to compare oncologic outcomes of these two different surgical procedures after preoperative CRT in low-lying LARC patients who were initially predicted to undergo APR, done without CRT.

## RESULTS

### Patient's characteristics

Out of 251 eligible patients that were included and received preoperative CRT, 122 patients (48.6%) underwent SPR while 129 patients (51.4%) had APR. The clinical characteristics of both groups were comparable, including age, gender, tumor size, and tumor staging (Table 1). However, pretreatment tumor location in SPR group was higher with mean of inferior tumor margin distance from the anal verge (DAV) of 5.0 cm, compared to only 3.3 cm in the APR group (*P*=0.001). Treatment parameters including preoperative and postoperative chemotherapy, dose of radiotherapy and the interval from the completion of CRT to surgery were also comparable between the two groups. The defunctioning stoma rate was 8.2% (10/122) in SPR group. Pathological details results are also shown in Table 1. As shown, patients in SPR group showed a better pathological downstaging after preoperative CRT, achieving significantly higher pCR rate compared to the APR group (32.8% vs 17.8%, *P*=0. 007). Besides, more regional lymph nodes were found in the rectal specimen of SPR group patients than those of the APR group (7 vs 5, *P* =0.030). All patients in the SPR group achieved negative distal resection margin (DRM) with the median length of 2.5 cm (range, 0.5 cm-5 cm).

**Table 1:** Clinical and pathological characteristics of patients with low-lying locally advanced rectal cancer

Variables	SPR group	APR group	*P* value
	n=122(%)	n=129(%)	
Gender			0.074
Male	76(62.3)	94(72.9)	
Female	41(33.7)	35(27.1)	
Median age, years (range)	57.5(15-78)	54(26-80)	0.056
Mean DAV, cm (SD)	5.0±0.9	3.3±1.5	<0.001
Mean size of tumor, cm (SD)	3.8±1.3	4.5±1.8	0.740
cT stage			0.231
3	79(64.8)	74(57.4)	
4	43(35.2)	55(42.6)	
cTNM stage			0.115
II	42(34.4)	57(44.2)	
III	80(65.6)	72(55.8)	
Preoperative chemotherapy regimen			0.057
XELOX	104(85.2)	97(75.2)	
FOLFOX	12(9.8)	26(20.2)	
Capecitabine	6(4.9)	6(4.7)	
Dose of radiotherapy, Gy (range)	46(30-50)	46(30-70)	0.121
Interval from the completion of CRT to surgery, weeks (range)	6.9(2.9-12.1)	6.7(1-24)	0.104
Mean size of tumor after CRT, cm (SD)	3.0±1.7	3.2±1.6	0.200
Tumor differentiation			0.227
Well and moderate	96(78.7)	93(72.1)	
Poor	26(21.3)	36(27.9)	
Median number of lymph nodes examined (range)	7(0-27)	5(0-37)	0.030
Median number of positive lymph nodes (range)	0(0-12)	0(0-8)	0.840
ypT stage			0.010
0-2	71(58.2)	54(41.9)	
3-4	51(41.8)	75(58.1)	
ypN stage			0.840
0	94(77.0)	98(76.0)	
1-2	28(23.0)	31(24.0)	
ypTNM stage			0.039
0	40(32.8)	23(17.8)	
I	23(19.0)	27(20.9)	
II	31(25.4)	48(37.2)	
III	28(23.0)	31(24.0)	
Achievement of pCR			0.007
Yes	40(32.8)	23(17.8)	
No	82(67.2)	106(82.2)	
Postoperative chemotherapy regimen			0.153
None	23(19.9)	21(16.3)	
Capecitabine	6(4.9)	15(11.6)	
XELOX or FOLFOX	93(76.2)	93(72.1)	
Median postoperative chemotherapy duration, weeks (range)	12(2-18)	12(2-18)	0.665

Abbreviations: APR: abdominoperineal resection, SPR: sphincterpreserving resection, DAV: inferior tumor margin distance from the anal verge, SD: standard deviation, cTNM stage: clinical tumor-node-metastasis classification, cT stage: clinical tumor stage, ypT stage: pathologic tumor stage after chemoradiotherapy, ypN stage: pathologic node stage after chemoradiotherapy, ypTNM stage: pathologic tumor-node-metastasis classification after chemoradiotherapy, pCR: pathologic complete response, CRT: chemoradiotherapy

### Postoperative complications

The morbidity of postoperative complications was 24.6% in SPR group, which was not significantly different from that of the APR group (20.9%, *P*=0.445). Postoperatively, dysphoria was the most observed complication (10.7%) in the SPR group. Other complications included anastomotic leakage (4.9%), anastomotic stricture (4.1%), intestinal obstruction (2.5%), postoperative pelvic bleeding (1.6%), and pelvic abscess (0.8%). On the other hand, poor wound healing was the most common complication in the APR group, observed in 16 (12.4%) patients. Other complications observed were intestinal obstruction 5 (3.9%), postoperative pelvic bleeding 2 (1.6%) and pelvic abscess found in only 1(0.8%) patient.

### Recurrence and survival

**Figure 1:**
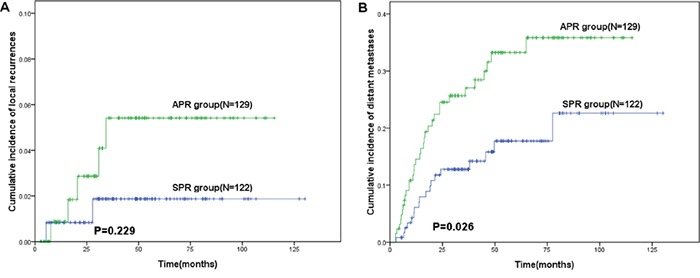
Kaplan–Meier curve of recurrences in patients with lower locally advanced rectal cancer treated with preoperative chemoradiotherapy followed by sphincter preserving resection (SPR) or abdominoperineal resection (APR) **A.** cumulative incidence of local recurrences. **B.** cumulative incidence of distant recurrences.

**Figure 2:**
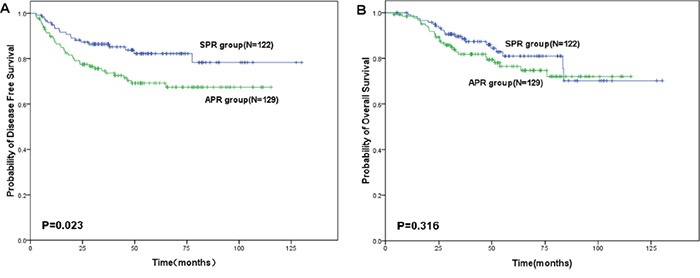
Survival of patients with lower locally advanced rectal cancer treated with preoperative chemoradiotherapy followed by sphincter preserving resection (SPR) or abdominoperineal resection (APR) **A.** 3-year disease free survival rate. **B.** 3-year overall survival rate.

The median duration of the follow-up was 48.6 months (range: 4–130 months). As shown in Table 2, local recurrence was observed in 2 patients (1.6%) of the SPR group and 5 (3.9%) of the APR group (*P*=0.297), while distant recurrence was documented in 18 patients (14.8%) of SPR group and 32 (24.8%) of APR group (*P*=0.046). Lung was the most common site of metastasis in both groups. The cumulative incidence of local recurrence within 3 years was 1.9 % in SPR group, which was not significantly different from that of the APR group (5.3%, *P*= 0.229, Figure 1A). However, cumulative incidence of distant recurrence within 3 years in APR group was almost two fold higher than that of the SPR group (23.7% vs 12.0%, *P*=0.026, Figure 1B). The estimated 3-year DFS was 86.4% for the SPR group, significantly higher than that of APR group (73.6%, *P*=0.023, Figure 2A), while the estimated 3-year OS rate was 89.6% for SPR group and 81.8% for APR group respectively (*P*=0.316, Figure 2B). Moreover, for SPR group patients, no significant difference in the 3-year DFS was found based on DRM (*P*=0.399, Table 3). Univariate analysis for the factors affecting the 3-year DFS revealed that surgical procedure, pathologic T and N stages were the significant prognostic factors (Table 3). These factors were further assessed by Cox proportional hazards model and surgical procedure was not found to be an independent prognostic factor (Table 4).

**Table 2:** Patterns of local and distant recurrence in the low-lying locally advanced rectal cancer patients after preoperative chemoradiotherapy and radical resection

Categories	Localization	SPR group	APR group	*P* value
		n=122(% of total)	n=129(% of total)	
Local recurrence				
Peripheral type		1(0.8)	4(3.1)	
Central type		1(0.8)	1(0.8)	
	Total	2(1.6)	5(3.9)	0.297
Distant recurrence				
Multiple organ		3(2.4)	4(3.2)	
Single organ	Lung	8(6.6)	11(8.5)	
	Liver	5(4.1)	9(7.0)	
	Bone	1(0.8)	2(1.6)	
	Peritoneum	1(0.8)	6(4.7)	
	Total	18(14.8)	32 (24.8)	0.046

Abbreviations: APR: abdominoperineal resection, SPR: sphincter preserving resection

**Table 3:** Univariate analysis of prognostic factors for 3-year disease free survival

Clinical factors	Cases	3-year DFS rate (%)	*P* value
Gender			0.371
Male	170	80.2	
Female	81	79	
Age, year			0.667
≤60	84	78.6	
>60	167	80.4	
DAV (cm)			0.075
≤3	75	71.1	
>3	176	83.4	
Preoperative chemoradiotherapy regimen			0.086
XELOX	201	81.9	
FOLFOX	38	80.7	
Capecitabine	12	41.7	
Dose of radiotherapy (Gy)			0.783
<50	188	79.9	
≥50	63	80.0	
postoperative chemotherapy duration (weeks)			0.559
<12	89	78.8	
≥12	162	80.4	
Surgical procedure			0.023
SPR	122	86.4	
APR	129	73.6	
Tumor differentiation			0.962
Well and moderate	189	79.4	
Poor	62	81.2	
ypT stage			<0.001
0-2	125	89	
3-4	126	70.8	
ypN stage			<0.001
0	192	86.7	
1-2	59	57	
DRM (cm)^a^			0.399
0-1	12	91.7	
1-2	47	91.1	
2-3	38	86.3	
3-4	14	71.4	
4-5	11	80	

Abbreviations: DFS: Disease Free Survival, APR: abdominoperineal resection, SPR: sphincter preserving resection, DAV: inferior tumor margin distance from the anal verge, ypT stage: pathologic tumor stage after chemoradiotherapy, ypN stage: pathologic node stage after chemoradiotherapy, DRM: distal resection margin.

a Patients only undergoing SPR

**Table 4:** Multivariate analysis of prognostic predictors for 3-year disease free survival

Variables	Cases	HR	95% CI for HR	*P* value
ypT stage				0.020
0-2	125	Reference	-	
3-4	126	2.090	1.123-3.889	
ypN stage				<0.001
0	192	Reference	-	
1-2	59	2.992	1.737-5.153	
Surgical procedure			0.075	
SPR	122	Reference	-	
APR	129	1.650	0.951-2.863	

Abbreviations: HR:Hazard ratio, APR: abdominoperineal resection, SPR: sphincter preserving resection, ypT stage: pathologic tumor stage after chemoradiotherapy, ypN stage: pathologic node stage after chemoradiotherapy

## DISCUSSION

Although APR is still a main procedure in the management of low-lying rectal cancer, the application of preoperative CRT combined with SPR has been becoming more widespread used [13, 14]. According to the final assessment of 251 patients with low-lying LARC, SPR was performed in 48.6% patients with significantly better 3-year DFS rate (*P*=0.023) and lower incidence of distant recurrence (*P*=0.026) compared to APR.

It was noted that response to preoperative CRT had an important role in increasing the possibility of preserving the anal sphincter in surgery [15, 16]. Preoperative CRT can help shrink the tumor to provide more operating space for surgical resection, which might be otherwise hard to perform [7]. In our study, we found that these patients who achieved pCR were more likely to undergo SPR than those who didn't (63.5% vs 43.6%, *P*=0.007). Added to that, due to tumor regression after preoperative CRT, DRM might be getting longer allowing enough distal rectal length for anastomosis. One concern of performing SPR for the low-lying rectal cancer is to ensure an adequate surgical distal resection margin to prevent local recurrence [17]. In general, DRM of 2 cm has been recommend for patients who have not received CRT [18], while it was demonstrated that after CRT, a DRM of at least 1 cm did not jeopardize oncological safety [19, 20]. Poricolo et al set 1 cm as the length of DRM for rectal cancer resection after CRT and observed no local recurrence within 48.8 months follow-up [19]. Kwak et al even suggested a distal margin of at least 5 mm with negative resection margin guaranteed oncological safety after CRT [20]. Our data also showed that once negative resection margin was achieved, 3-year DFS seemed not to be correlated with DRM after preoperative CRT (*P*=0.399). Therefore, preoperative CRT can provide a shorter resection margin without increasing the risk of local and distant recurrence, which makes colorectal anastomosis easier.

Although the oncologic outcome was a highlight of selecting the feasible surgical procedure for low-lying LARC, there were limited studies comparing oncologic outcomes between SPR and APR following CRT [12, 21, 22]. Huh et al reported that there were no significant difference in the overall recurrence rate (20.9% vs 20.5%, *P* = 0.956) and 5-year OS(70.8% vs 62.9%, *P*= 0.189) [12]. On the contrary, Weiser et al noted that APR compromise local control and recurrence-free survival. They showed that 5-years recurrence-free survival for the stapled anastomosis, intersphincteric resection, and APR groups were 85%, 83%, and 47% respectively (P< 0.001) [21]. Kim et al also reported that patients who underwent APR had higher 5-year local recurrence (22.0% vs. 11.5%, *P* = 0.028) and lower 5-year cancer-specific survival rate (52.9% vs. 71.1%, *P* = 0.030) than those who underwent SPR [22]. Similarly, our results showed that even though incidence of local recurrence was comparable between the two groups (1.9% vs 5.3%, *P* = 0.229), the distant recurrence in APR was significantly higher than the SPR group (23.7% vs 12.0%, P=0.026). In addition, patients in APR group exhibited worse 3-year DFS than those in the SPR group (73.6% vs 86.4%, *P*=0.023). To explain the common results that APR delivered a worse recurrence control and worse oncologic outcome, the postoperative pathologic factors of patients should be taken into consideration. Sphincter preservation tends to be considered more in the patients with a significant pathologic response to preoperative CRT. On the other hand, older patients, patients with poor tumor differentiation and less response tumor were more likely to undergo APR. These prognostic high-risk factors were also associated with higher recurrence and worse survival [21, 23]. In this current study, pathologic T and N stage were the independent predictors for 3-year DFS (*P* =0.020 and *P*<0.001). Since achievement of pCR after preoperative CRT is associated with greatly improved long-term outcome in LARC [24, 25], a higher pCR rate in SPR group might account for the better oncologic outcome in comparison with the APR group (32.8% vs 17.8%, *P*=0.007).

Several potential limitations should be acknowledged. First of all, selective bias of dividing the patients into different groups was unavoidable due to the retrospective nature of this study. As we have mentioned above, patients with lower tumor location (DAV < 3cm) were only selected for APR, which resulted in the bias of tumor location in these two groups. However, with careful planning and a multidisciplinary approach, rectal cancer height did not influence the oncological outcome [26, 27]. Similarly, we found tumor location had no significant impact on DFS in the current study, as showed in Table 3. Furthermore, the surgeon specialization and technical expertise have been considered as important factors of the surgical procedure decision for low-lying LARC [28]. Since quality of life was another important long-term outcome of rectal cancer after surgery, we also failed to evaluate it in our study. This limitation may have led to underestimate the quality of life after CRT followed by SPR or APR influencing the oncologic survival. Moreover, we also realized that the insufficient follow-up time was unable to measure the 5-year OS for patients. Despite of those limitations mentioned above, our study indeed provided the critical information for the optimal strategy for treating low-lying LARC patients.

In conclusion, preoperative CRT provides an opportunity for those who initially deserve APR to undergo SPR without compromising oncologic survival. SPR can be a practicable alternative for the treatment of low-lying LARC patients who response well to preoperative CRT, to approach the concurrent requirement of adequate oncological control along with sphincter functional preservation.

## PATIENTS AND METHODS

### Patient selection

This retrospective cohort study enrolled a total of 251 consecutive LARC patients who underwent preoperative CRT followed by radical resection at Sun Yat-sen University Cancer center between March 2003 and November 2012. All patients met the following inclusion criteria: (1) Histological diagnosed rectal adenocarcinoma; (2) Inferior tumor margin within 6 cm from the anal verge before CRT; (3) T3-4 or N+ disease of pretreatment; (4) Radical resection with either SPR or APR; (5) No metastatic disease before and during preoperative treatment; (6) No other active malignancy (excluding skin basal cell carcinoma). Pretreatment staging was performed by endorectal ultrasound (EUS), chest and abdominopelvic computed tomography scanning (CT), and/or pelvic magnetic resonance imaging (MRI). Tumor location was confirmed both by colonoscopy and digital rectal examination. The eligible patients were assigned into two different groups according to the surgical procedures they had received, either SPR group or APR group. Overall data including the general clinical and tumor characteristics, treatment parameters, postoperative complications were recorded in detail. The study was done in accordance with the ethical standards of the World Medical Association Declaration of Helsinki. A Waiver of Informed Consent was requested and the study approval was obtained from independent ethics committees at Sun Yat-Sen University Cancer Center.

### Treatment

Radiation therapy was administered using three dimensional conformal radiation therapy (3D-CRT) or intensity-modulated radiation therapy (IMRT). All patients were scheduled a total irradiation dose of 46.0–50.40 Gy to the pelvic area, delivered in fractions of 1.8 Gy or 2.0 Gy daily on five consecutive days per week during 5-6 weeks. The clinical target volume included macroscopic tumor, entire mesorectum, and pararectal lymph nodes, together with internal iliac, anal sphincter complex, promontory and pre-sacral lymph nodes up to the level of the fifth lumbar vertebra. XELOX regimen (oxaliplatin was administered intravenously 130 mg/m^2^ on days 1 and capecitabine was administered orally 1,000 mg/m^2^ twice daily on days 1-14 for 3 week-cycle), FOLFOX regimen (oxaliplatin 85 mg/m^2^ and leucovorin 400 mg/m^2^ was administered intravenously over 2 hours on the first day, 5-fluorouracil was injected intravenously 400 mg/m^2^ on the first day and then administered 2400 mg/m^2^ by continuous intravenous infusion for 46 hours for 2 week-cycle) or oral capecitabine (oral capecitabine 825 mg/m^2^ was given twice daily during radiotherapy without weekend breaks) was alternatively delivered concurrently with neoadjuvant radiotherapy.

Radical surgery was planned to be performed between 6 to 8 weeks after the completion of preoperative radiotherapy. All surgical operations were performed by experienced surgeons. The decision of surgical procedure was based on tumor location, pelvic space and the extent of anal sphincter invasion, while TME was performed whenever possible. For the unsatisfactory colorectal anastomosis, a diverting loop ileostomy was required.

Pathological assessment of the resected specimens was confirmed according to tumor-node-metastasis classification (TNM) by two independent pathologists. Pathologic complete response (pCR) was defined as follow: the absence of viable tumor cells with only fibrotic masses or acellular mucin pools present in area of primary tumor and lymph nodes [29]. Distal margins were considered positive once microscopic tumor was identified within 1 mm of resection [30]. Oxaliplatin based adjuvant chemotherapy was recommended.

### Endpoints and follow-up

The primary endpoint was 3-year DFS, while secondary endpoints included OS, cumulative incidence of recurrences and postoperative complications. All patients were evaluated through subsequent visits every 3 months for 2 years and then semiannually until 5 years postoperatively. At each follow-up visit, evaluation included physical examination, serum carcinoembryonic antigen (CEA) level, chest X-ray and abdominal ultrasound. Chest CT, abdominal/pelvic MRI, and colonoscopy were performed annually. Recurrence was confirmed by histological examination whenever possible or after assessment by a multidisciplinary treatment group. Recurrence in pelvis was defined as local recurrence and recurrence outside the pelvis was considered as distant metastasis.

### Statistical analysis

All data were analyzed using Statistical Package for the Social Sciences (SPSS, version 17.0, Chicago, IL). Quantitative variables were presented as mean (standard deviation) or median (range) and then compared based on the Student t-test or Mann-Whitney U test which ever appropriate. Qualitative variables were given as percentage and compared by applying Chi square test or Fisher's exact, as appropriate. Kaplan-Meier methodology was applied to calculate DFS, OS and cumulative incidences of recurrence by outlining the survival curve. Log-rank test was applied to distinguish the difference between groups. Univariate analysis of prognostic factors was also evaluated by log-rank test. Variables proved statistical significance in the univariate survival analysis were further assessed by Cox proportional hazards model, which was generated with forward stepwise selection of variables. All tests were two-tailed, in which P value < 0.05 was considered to be statistically significant.

## References

[1] Chessin DB, Guillem JG (2005). Abdominoperineal resection for rectal cancer: historic perspective and current issues. Surgical oncology clinics of North America.

[2] Bosset JF, Collette L, Calais G, Mineur L, Maingon P, Radosevic-Jelic L, Daban A, Bardet E, Beny A, Ollier JC, Trial ERG (2006). Chemotherapy with preoperative radiotherapy in rectal cancer. The New England journal of medicine.

[3] Sauer R, Becker H, Hohenberger W, Rodel C, Wittekind C, Fietkau R, Martus P, Tschmelitsch J, Hager E, Hess CF, Karstens JH, Liersch T, Schmidberger H, Raab R, German Rectal Cancer Study G (2004). Preoperative versus postoperative chemoradiotherapy for rectal cancer. The New England journal of medicine.

[4] Rodel C, Martus P, Papadoupolos T, Fuzesi L, Klimpfinger M, Fietkau R, Liersch T, Hohenberger W, Raab R, Sauer R, Wittekind C (2005). Prognostic significance of tumor regression after preoperative chemoradiotherapy for rectal cancer. J Clin Oncol.

[5] Park IJ, You YN, Agarwal A, Skibber JM, Rodriguez-Bigas MA, Eng C, Feig BW, Das P, Krishnan S, Crane CH, Hu CY, Chang GJ (2012). Neoadjuvant treatment response as an early response indicator for patients with rectal cancer. J Clin Oncol.

[6] Habr-Gama A, Perez RO, Kiss DR, Rawet V, Scanavini A, Santinho PM, Nadalin W (2004). Preoperative chemoradiation therapy for low rectal cancer. Impact on downstaging and sphincter-saving operations. Hepato-gastroenterology.

[7] Crane CH, Skibber JM, Feig BW, Vauthey J-N, Thames HD, Curley SA, Rodriguez-Bigas MA, Wolff RA, Ellis LM, Delclos ME, Lin EH, Janjan NA (2003). Response to preoperative chemoradiation increases the use of sphincter-preserving surgery in patients with locally advanced low rectal carcinoma. Cancer.

[8] Russell MM, Ganz PA, Lopa S, Yothers G, Ko CY, Arora A, Atkins JN, Bahary N, Soori GS, Robertson JM, Eakle J, Marchello BT, Wozniak TF, Beart RW, Wolmark N (2015). Comparative effectiveness of sphincter-sparing surgery versus abdominoperineal resection in rectal cancer: patient-reported outcomes in National Surgical Adjuvant Breast and Bowel Project randomized trial R-04. Ann Surg.

[9] Maslyankov S, Penchev D, Todorov G, Vladov N (2015). A Meta-Analysis of Quality of Life Estimated by Questionnaires of the European Organization for Research and Treatment of Cancer (EORTC) after Rectal Cancer Surgery. Chirurgia.

[10] Engel J, Kerr J, Schlesinger-Raab A, Eckel R, Sauer H, Holzel D (2003). Quality of life in rectal cancer patients: a four-year prospective study. Ann Surg.

[11] Do L, Syed N, Puthawala A, Azawi S, Shbeeb I, Gong IY (2011). Low-lying rectal cancer with anal canal involvement: abdominoperineal or low anterior resection after neoadjuvant chemoradiotherapy. Gastrointest Cancer Res.

[12] Huh J, Jung E, Park Y, Lee K, Sohn S-K (2008). Sphincter-Preserving Operations Following Preoperative Chemoradiation: An Alternative to Abdominoperineal Resection for Lower Rectal Cancer?. World J Surg.

[13] Wasserberg N, Kundel Y, Purim O, Keidar A, Kashtan H, Sadot E, Fenig E, Brenner B (2014). Sphincter preservation in distal CT2N0 rectal cancer after preoperative chemoradiotherapy. Radiation oncology.

[14] Janjan NA, Khoo VS, Abbruzzese J, Pazdur R, Dubrow R, Cleary KR, Allen PK, Lynch PM, Glober G, Wolff R, Rich TA, Skibber J (1999). Tumor downstaging and sphincter preservation with preoperative chemoradiation in locally advanced rectal cancer: the M D Anderson Cancer Center experience. International journal of radiation oncology biology physics.

[15] Crane CH, Skibber JM, Feig BW, Vauthey JN, Thames HD, Curley SA, Rodriguez-Bigas MA, Wolff RA, Ellis LM, Delclos ME, Lin EH, Janjan NA (2003). Response to preoperative chemoradiation increases the use of sphincter-preserving surgery in patients with locally advanced low rectal carcinoma. Cancer.

[16] Luna-Perez P, Rodriguez-Ramirez S, Rodriguez-Coria DF, Fernandez A, Labastida S, Silva A, Lopez MJ (2001). Preoperative chemoradiation therapy and anal sphincter preservation with locally advanced rectal adenocarcinoma. World J Surg.

[17] Komori K, Kanemitsu Y, Ishiguro S, Shimizu Y, Sano T, Ito S, Abe T, Senda Y, Misawa K, Ito Y, Uemura N, Kato T (2012). Adequate length of the surgical distal resection margin in rectal cancer: from the viewpoint of pathological findings. American journal of surgery.

[18] Shimada Y, Takii Y, Maruyama S, Ohta T (2011). Intramural and mesorectal distal spread detected by whole-mount sections in the determination of optimal distal resection margin in patients undergoing surgery for rectosigmoid or rectal cancer without preoperative therapy. Diseases of the colon and rectum.

[19] Pricolo VE, Abodeely A, Resnick M (2010). Distal margins in radical resections for rectal cancer after chemoradiation therapy: how short is long enough?. Digestive surgery.

[20] Kwak JY, Kim CW, Lim SB, Yu CS, Kim TW, Kim JH, Jang SJ, Kim JC (2012). Oncologically safe distal resection margins in rectal cancer patients treated with chemoradiotherapy. Gastrointest Surg.

[21] Weiser MR, Quah HM, Shia J, Guillem JG, Paty PB, Temple LK, Goodman KA, Minsky BD, Wong WD (2009). Sphincter preservation in low rectal cancer is facilitated by preoperative chemoradiation and intersphincteric dissection. Ann Surg.

[22] Kim JS, Hur H, Kim NK, Kim YW, Cho SY, Kim JY, Min BS, Ahn JB, Keum KC, Kim H, Sohn SK, Cho CH (2009). Oncologic outcomes after radical surgery following preoperative chemoradiotherapy for locally advanced lower rectal cancer: abdominoperineal resection versus sphincter-preserving procedure. Ann Surg Oncol.

[23] Reshef A, Lavery I, Kiran RP (2012). Factors associated with oncologic outcomes after abdominoperineal resection compared with restorative resection for low rectal cancer: patient- and tumor-related or technical factors only?. Diseases of the colon and rectum.

[24] de Campos-Lobato LF, Stocchi L, da Luz Moreira A, Geisler D, Dietz DW, Lavery IC, Fazio VW, Kalady MF (2011). Pathologic complete response after neoadjuvant treatment for rectal cancer decreases distant recurrence and could eradicate local recurrence. Ann Surg Oncol.

[25] Maas M, Nelemans PJ, Valentini V, Das P, Rodel C, Kuo LJ, Calvo FA, Garcia-Aguilar J, Glynne-Jones R, Haustermans K, Mohiuddin M, Pucciarelli S, Small W (2010). Long-term outcome in patients with a pathological complete response after chemoradiation for rectal cancer: a pooled analysis of individual patient data. The Lancet Oncology.

[26] Bhangu A, Rasheed S, Brown G, Tait D, Cunningham D, Tekkis P (2014). Does rectal cancer height influence the oncological outcome?. Colorectal Dis.

[27] Diaz-Gonzalez JA, Calvo FA, Cortes J, Garcia-Sabrido JL, Gomez-Espi M, Del Valle E, Munoz-Jimenez F, Alvarez E (2006). Prognostic factors for disease-free survival in patients with T3-4 or N+ rectal cancer treated with preoperative chemoradiation therapy surgery and intraoperative irradiation. International journal of radiation oncology biology physics.

[28] Cong ZJ, Hu LH, Xing JJ, Zhang W, Fu CG, Yu ED, Zhong M (2014). Risk factors associated with sphincter-preserving resection in patients with low rectal cancer. International surgery.

[29] Mandard AM, Dalibard F, Mandard JC, Marnay J, Henry-Amar M, Petiot JF, Roussel A, Jacob JH, Segol P, Samama G (1994). Pathologic assessment of tumor regression after preoperative chemoradiotherapy of esophageal carcinoma. Clinicopathologic correlations. Cancer.

[30] Quirke P, Durdey P, Dixon MF, Williams NS (1986). Local recurrence of rectal adenocarcinoma due to inadequate surgical resection. Histopathological study of lateral tumour spread and surgical excision. Lancet.

